# Electrochemical Determination of Interaction between SARS-CoV-2 Spike Protein and Specific Antibodies

**DOI:** 10.3390/ijms23126768

**Published:** 2022-06-17

**Authors:** Maryia Drobysh, Viktorija Liustrovaite, Ausra Baradoke, Alma Rucinskiene, Almira Ramanaviciene, Vilma Ratautaite, Roman Viter, Chien-Fu Chen, Ieva Plikusiene, Urte Samukaite-Bubniene, Rimantas Slibinskas, Evaldas Ciplys, Martynas Simanavicius, Aurelija Zvirbliene, Indre Kucinskaite-Kodze, Arunas Ramanavicius

**Affiliations:** 1 NanoTechnas—Center of Nanotechnology and Materials Science, Faculty of Chemistry and Geosciences, Vilnius University, 03225 Vilnius, Lithuania; mariadrobysh@gmail.com (M.D.); viktorijaliustrovaite@gmail.com (V.L.); alma@chi.lt (A.R.); almira.ramanaviciene@chf.vu.lt (A.R.); vilma.ratautaite@ftmc.lt (V.R.); ieva.plikusiene@chgf.vu.lt (I.P.); urte.samukaite-bubniene@chf.vu.lt (U.S.-B.); 2 State Research Institute Center for Physical and Technological Sciences, LT-10257 Vilnius, Lithuania; ausra.baradoke@ftmc.lt; 3 State Research Institute Center of Innovative Medicine, LT-08406 Vilnius, Lithuania; 4 Institute of Atomic Physics and Spectroscopy, University of Latvia, LV-1004 Riga, Latvia; roman.viter@lu.lv; 5 Center for Collective Use of Research Equipment, Sumy State University, 40000 Sumy, Ukraine; 6 Institute of Applied Mechanics, National Taiwan University, Taipei City 106, Taiwan; stevechen@iam.ntu.edu.tw; 7 Institute of Biotechnology, Life Sciences Center, Vilnius University, LT-10257 Vilnius, Lithuania; rimantas.slibinskas@bti.vu.lt (R.S.); evaldas.ciplys@bti.vu.lt (E.C.); martynas.simanavicius@bti.vu.lt (M.S.); aurelija.zvirbliene@bti.vu.lt (A.Z.); indre.kodze@bti.vu.lt (I.K.-K.)

**Keywords:** COVID-19, SARS-CoV-2 coronavirus, electrochemical immunosensor, electrochemical impedance spectroscopy (EIS), cyclic voltammetry (CV), self-assembled monolayer (SAM), antigen-antibody complex, spike proteins (rSpike), specific antibodies, serological diagnosis

## Abstract

The serologic diagnosis of coronavirus disease 2019 (COVID-19) and the evaluation of vaccination effectiveness are identified by the presence of antibodies specific to severe acute respiratory syndrome coronavirus 2 (SARS-CoV-2). In this paper, we present the electrochemical-based biosensing technique for the detection of antibodies specific to the SARS-CoV-2 proteins. Recombinant SARS-CoV-2 spike proteins (rSpike) were immobilised on the surface of a gold electrode modified by a self-assembled monolayer (SAM). This modified electrode was used as a sensitive element for the detection of polyclonal mouse antibodies against the rSpike (anti-rSpike). Electrochemical impedance spectroscopy (EIS) was used to observe the formation of immunocomplexes while cyclic voltammetry (CV) was used for additional analysis of the surface modifications. It was revealed that the impedimetric method and the elaborate experimental conditions are appropriate for the further development of electrochemical biosensors for the serological diagnosis of COVID-19 and/or the confirmation of successful vaccination against SARS-CoV-2.

## 1. Introduction

Biosensors have piqued the interest of many researchers in recent years, particularly in the realm of healthcare. They are distinguished by their rapid response time, ultrasensitive detection of biomolecules, and the ability to be miniaturized for a portable application while needing minimal sample processing when compared to conventional analytical procedures. The primary principle underlying biosensing devices is the conversion of biotarget detection into an analytical signal for further analysis. A variety of molecules including enzymes [1,2], proteins [3,4], antibodies [5,6], and nucleic acids [7,8] can be used as target biomolecules, with electrochemical [5,9,10], optical [11], piezoelectric [12], surface plasmon resonance [13], and other methods being commonly used for the analytical signal registration.

Coronavirus disease 2019 (COVID-19) diagnostic techniques based on biosensors are generally classified into two categories depending on the target compounds: molecular and serological [14]. The serological type is based on the detection of the affinity interaction between antigens and specific antibodies. The determination of specific antibodies allows one to define the stage of the disease and evaluate the immune response toward severe acute respiratory syndrome coronavirus 2 (SARS-CoV-2) infection. The spike (S) protein is commonly used as the antigen in serological tests [15]. The SARS-CoV-2 structural S-protein is a transmembrane homotrimer that is required for viral adherence and penetration of a host cell [16,17].

Due to their low cost, simplicity, and availability for mass production, electrochemical biosensors are widely investigated in the biomedical applications [18,19,20,21].

However, electrochemical-based biosensors for the diagnosis of COVID-19 are still facing some challenges in order to be commercialised and further research is in high demand [22].

Recently, for electrochemical detection of SARS-CoV-2-related proteins, various electrochemical methods to evaluate analytical signals were reported [23,24,25,26,27,28,29]. The antibodies against SARS-CoV-2 were detected using differential pulse voltammetry [30], chronoamperometry [31], pulsed amperometric detection [4,7], square wave voltammetry [10], cyclic voltammetry (CV) [32,33], and electrochemical impedance spectroscopy (EIS) [5,9,34].

In this paper, we investigate an electrochemical-based approach for the detection of polyclonal mouse antibodies against the recombinant SARS-CoV-2 S-protein (rSpike). EIS and CV were chosen as the analytical methods for evaluating the antigen-antibody interaction taking place on the working gold electrode surface since they were both simple and straightforward. It is believed that the antigen-antibody complex produces a blocking layer in the biosensing system, which causes the electron transfer resistance to increase.

Due to the low amplitude of perturbation from steady-state, the EIS-based system allows non-destructive direct sensing of target biomolecules without employing enzyme labels [35]. CV is used for the evaluation of electrochemical properties of analyte solutions as well as the blockage of the electrode surface [36].

Because the target rSpike is detected on the working electrode’s surface, it is necessary to design the surface with proper protein recognition characteristics. For this purpose, a self-assembled monolayer (SAM) is commonly used; among these, -COOH terminated SAM was shown as one of the most appropriate for specific and stable SARS-CoV-2 S-protein immobilisation [37]. 11-mercaptoundecanoic acid (11-MUA), based on alkanethiols, forms a firm and dense film and makes it possible to observe the kinetics of mediated electron passage [38]. In our previous work [29], covalent immobilization of the SARS-CoV-2 S-protein and its affinity interaction with specific antibodies against SARS-CoV-2 virus proteins in blood serum patient samples after coronavirus disease 2019 (COVID-19) (anti-rSpike) were evaluated. The anti-rSpike was quantified using CV and EIS methods, giving the limit of detection values of 2.53 nM and 1.99 nM, respectively. This research aimed to investigate the event of antigen-antibody complex formation occurring on the working electrode surface by EIS with an additional assessment of the examined surface blockage by CV. The findings of this study will serve as the foundation for the design of a biosensor powered by other electrochemical technologies.

## 2. Experimental

### 2.1. Chemicals and Other Materials

11-mercaptoundecanoic acid (11-MUA) (98%, CAS# 71310-21-9) and methanol (MeOH) (≥99%, CAS# 67-56-1) were obtained from Sigma–Aldrich (Steinheim, Germany), N-hydroxysuccinimide (NHS) (98%, CAS# 6066-82-6) and N-(3-dimethylaminopropyl)-N’-ethyl-carbodiimide hydrochloride (EDC) (≥99.0%, CAS# 25952-53-8) were purchased from Alfa Aesar (Karlsruhe, Germany), alumina suspension (grain diameter 0.3 µm) was received from Buehler (Lake Bluff, IL, USA). Baltymas (Vilnius, Lithuania) developed the recombinant SARS-CoV-2 spike protein (rSpike). In accordance with the protocol outlined hereunder, polyclonal antibodies against rSpike (anti-rSpike) were produced. Complete Freund’s adjuvant (CFA) and Incomplete Freund’s adjuvant (IFA) were purchased from Thermo Fisher Scientific (USA). Ammonium sulfate (CAS# 7783-20-2, purity >99.5%) was obtained from Carl Roth (Germany). K_3_Fe(CN)_6_ (≥99.0%, CAS# 13746-66-2), K_4_Fe(CN)_6_ (≥99.0%, CAS# 14459-95-1), NaBH_4_ (≥98.0%, CAS# 16940-66-2), NaCl (≥99.0%, CAS# 7647-14-5), KCl (≥99.0%, CAS# 7447-40-7), NaH_2_PO_4_ (≥99.0%, CAS# 7558-80-7), K_2_HPO_4_ (≥98.0%, CAS# 7758-11-4). Deionized water was used to prepare all aqueous solutions. All reagents were of analytical-reagent grade and were used as received from the producers unless otherwise noted.

All electrochemical measurements were carried out in 0.1 M phosphate buffer saline solution (PBS), pH 7.4 with the presence of 2 mM K_4_Fe(CN)_6_/K_3_Fe(CN)_6_ ([Fe(CN)_6_]^3−/4−^). PBS was prepared by dissolving 0.137 M NaCl, 0.01 M NaH_2_PO_4_, 0.0027 M KCl, and 0.0018 M of KH_2_PO_4_ in deionized water.

### 2.2. Protocol of Protein Purification

Hamster CHO cells obtained from Thermo Fisher Scientific (Waltham, MA, USA) (cat. no. A29127) were used for the secretion of rSpike protein. The gene, which encodes the SARS-CoV-2 Spike ectodomain including amino acids (aa) 1-1208, (UniProtKB sequence accession number: P0DTC2 (SPIKE_SARS2)) was obtained from General Biosystems (USA). This gene was integrated into the expression vector pCAGGS (Creative Biogene, cat. no. VET1375) through the restriction sites NotI and XhoI, which are added at 5′ and 3′ ends of this gene, correspondingly. These expression constructs contain these parts: (i) full-length rSpike ectodomain (aa 1–1208) without transmembrane and cytoplasmic aa, (ii) furin cleavage site ‘RRAR’ mutated to “GSAS”, (iii) C-terminal GSN4 trimerisation motif fused to protein sequence, (iv) thrombin cleavage site, and (v) Strep-tag II and His6-tag. Two mutations (K986P and V987P) were introduced into the rSpike sequence to stabilize the trimer in the pre-fusion conformation [39]. The rSpike protein was generated in CHO cells (cat. no. A29133) grown in ExpiCHO Expression System purchased from Thermo Fisher Scientific’s (Vilnius, Lithuania). The Max Titer protocol was developed by Thermo Fisher Scientific (Vilnius, Lithuania) and was applied for protein transfection and expression procedures. Transfection lasted nine days, then cells were harvested from cultivation media and under refrigeration were centrifuged at 5000 g for 30 min. Then, supernatant was filtered using a filter that contained cavities of 0.22-µm diameter. The supernatant was condensed and then dissolved in 50 mM PBS, pH 8.0, containing 10 mM imidazole and 300 mM NaCl through tangential ultrafiltration by TFF cassette, which was supported with 100 kDa cutoff membranes (cat. no. VF20P) from Sartorius Stedim Biotech (Göttingen, Germany). The protein solution was deposited onto Ni-NTA resin from Super Flow (Qiagen, Germantown, MD, USA). Next, non-specifically bound proteins were removed using the chromatography column using a ‘Lysis’ buffer with 75 mM imidazole. More tightly bound proteins were eluted by a ‘gradient solution’ containing 75–250 mM imidazole. The fractions containing purified rSpike glycoprotein were pooled and dialyzed against 10 mM PBS, pH 7.4, containing 3 mM of KCl and 140 mM of NaCl. Then, the solution was diluted down to 1.0 mg/mL, filtered, and separated into small samples that were stored in a frozen state before use in the experiments. SDS-PAGE electrophoresis was applied for the determination of rSpike protein purity, which was ~90%. Anti-rSpike protein was produced by BALB/c mice. Female mice were subcutaneously immunised four times (at intervals of 28 days) with 50 µg of rSpike protein. The antigen was emulsified by complete Freund’s adjuvant during the first injection and/or incomplete adjuvant during the second injection, respectively. The third and fourth immunisations were performed via antigen diluted in PBS. The mouse was sacrificed by applying cervical dislocation four days after the final immunisation. Then, whole blood samples were collected from the chest cavity. The collected blood was centrifuged at 300× *g* for 10 min, and the resulting supernatant was diluted by saturated ammonium sulfate solution at a ratio of 1:1. This solution containing polyclonal antibodies was incubated at 4 °C for 16 h. The fraction of immunoglobulin G was separated by centrifugation at 12,000× *g* for 10 min. The collected precipitate was re-dissolved in 10 mM PBS, pH 7.4, and the solution was then mixed with a similar volume of saturated ammonium sulfate solution. In this solution, total protein concentration was determined spectrophotometrically. Mice used for the immunisation experiments were obtained from the breeding colony of Life Sciences Center of Vilnius University (Vilnius, Lithuania). Animal maintenance and experimental protocols were performed in accordance with FELASA guidelines and Lithuanian and European legislation. Permission No. G2-117 for the generation of polyclonal and monoclonal antibodies was issued by the State Food and Veterinary Service, Vilnius, Lithuania.

### 2.3. Preparation of Gold Electrode Surface

The geometrical area of the chemically pure (99.9%) square gold (Au) electrode was 1 cm^2^. The surface of the Au electrode was mechanically polished using an alumina suspension. After polishing, the Au surface was cleaned in an ultrasonic bath (EMAG Emmi-40 HC) with water for 10 min. Subsequently, the electrode was kept in 0.5 M NaBH_4_ solution for 10 min (H_2_O/MeOH, *v*/*v*, 1:1) [40]. The working Au electrode was reused after each experiment, going through the same steps described in this manuscript.

### 2.4. The Activation of 11-MUA Based SAM and Covalent Immobilisation of the rSpike Protein

**Figure 1:**
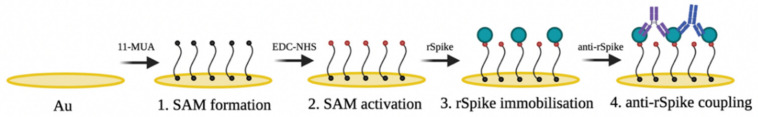
Schematic representation of experimental stages: (1) 11-MUA SAM layer formation on the Au electrode (Au/SAM); (2) SAM activation by EDC-NHS mixture; (3) rSpike immobilisation and formation of Au/SAM/rSpike sensing structure; (4) affinity interaction of anti-rSpike with immobilised rSpike.

To achieve this goal, the Au electrode was incubated in 1 mM 11-MUA solution in MeOH at 24 °C for 18 h (Figure 1, step 1). Following incubation, the electrode was rinsed with MeOH to remove the remaining 11-MUA and dried with N_2_. SAM, which was formed on the Au electrode surface (Au/SAM) and activated by the EDC-NHS mixture. The reaction of 11-MUA carboxyl groups with a mixture of 0.04 M EDC and 0.01 M NHS in water resulted in functionally active NHS-esters (Figure 1, step 2). The activation procedure was performed in the dark for 20 min. After activating the carboxyl functional groups, the electrode was incubated in 1 mL of 50 g/mL rSpike in PBS solution for 45 min at room temperature. rSpike was covalently attached through primary amine functional groups (Figure 1, step 3). The remains of the active esters were deactivated with 1 mM EA solution, pH 8.5 for 10 min (Figure 1, step 4). Then, 1 mL of 50 µg/mL anti-rSpike solution was added and the affinity interaction of antibodies specific to rSpike was performed at room temperature for 1 h. After the incubation, the formed Au/SAM/rSpike/anti-rSpike structure was washed with PBS solution and utilised for further electrochemical measurements. The formed Au/SAM, Au/SAM/EDC-NHS, and Au/SAM/rSpike electrodes were used in all subsequent electrochemical experiments. Au/SAM/rSpike electrodes were used for the detection of antibodies specific towards rSpike.

### 2.5. Electrochemical Measurements

The bare Au electrode, Au/SAM, Au/SAM/EDC-NHS, and Au/SAM/rSpike electrodes were electrochemically characterised using the potentiostat/galvanostat AUTOLAB TYPE III (Metrohm, Netherlands) operated by FRA2-EIS ECO-Chemie software (Utrecht, Netherlands). Experiments before and after incubation stages were performed in PBS, pH 7.4, with 2 mM of [Fe(CN)_6_]^3−/4−^ to eliminate the impact of the electrolyte composition. The experiments were carried out in the three-electrode electrochemical cell, which included the Au-based electrode (Au, Au/SAM, Au/SAM/EDC-NHS, and Au/SAM/rSpike) working electrode, platinum (Pt) counter electrode, and as a reference electrode, Ag/AgCl in 3M KCl (Ag/AgCl_(3M KCl)_) microelectrode (IS-AG/AGCL.AQ.RE) (ItalSens, Netherland) was used. CV and EIS techniques were used to characterise the electrochemical properties of bare Au, Au/SAM, Au/SAM/EDC-NHS, and Au/SAM/rSpike electrodes at diverse steps of modification. At a scan rate of 50 mV/s, CV measurements were carried out in the potential window of 0 to + 0.4 V vs Ag/AgCl_(3M KCl)_. A perturbation amplitude of 10 mV was used to register the EIS in the frequency range between 0.1 Hz and 100 kHz.

## 3. Results and Discussion

### Electrochemical Characterisation

**Figure 2:**
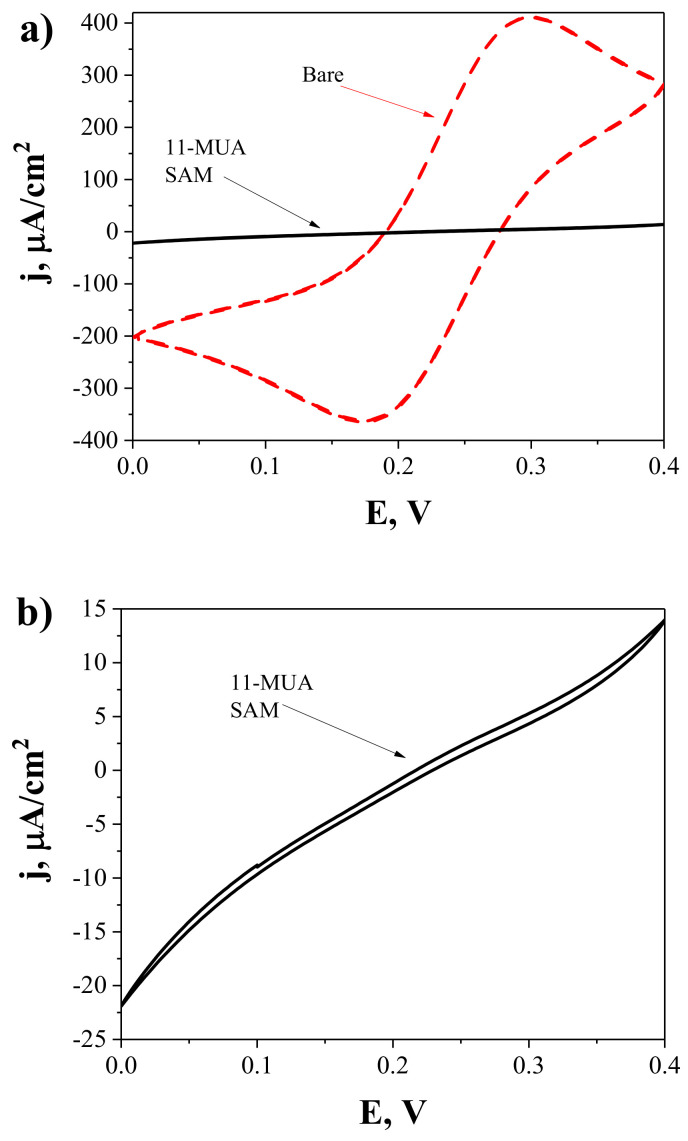
(**a**) Cyclic voltammograms of the bare Au electrode (dashed line) and Au/SAM electrode after the formation of 11-MUA SAM (solid line). (**b**) Scaled cyclic voltammogram of the Au/SAM electrode. Measurements were performed in PBS while adding 2 mM of [Fe(CN)_6_]^3−/4−^. Potential scans range from 0 to +0.4 V vs Ag/AgCl_(3M KCl)_ at 50 mV/s.

CV and EIS measurements were used to characterise the Au electrode before and after 11-MUA SAM formation. Using a [Fe(CN)_6_]^3−/4−^ couple as a redox probe and analysing the oxidation/reduction peaks of the resulting cyclic voltammogram, the influence of each stage of the surface modification of the working electrode on conductivity was investigated. Figure 2 shows the cyclic voltammogram of the Au electrode before and after the formation of the 11-MUA monolayer. On the electrode surface, long-chain thiols (*n* = 10) create a very stable and well-organised monolayer, which thus acts as an ionic insulator on a gold electrode. SAM has a lower defect rate and a higher fraction coverage rate [41]. As a result, additional 11-MUA molecules can obstruct the electron transfer pathway, considerably suppressing the current response (Figure 2b).

**Figure 3:**
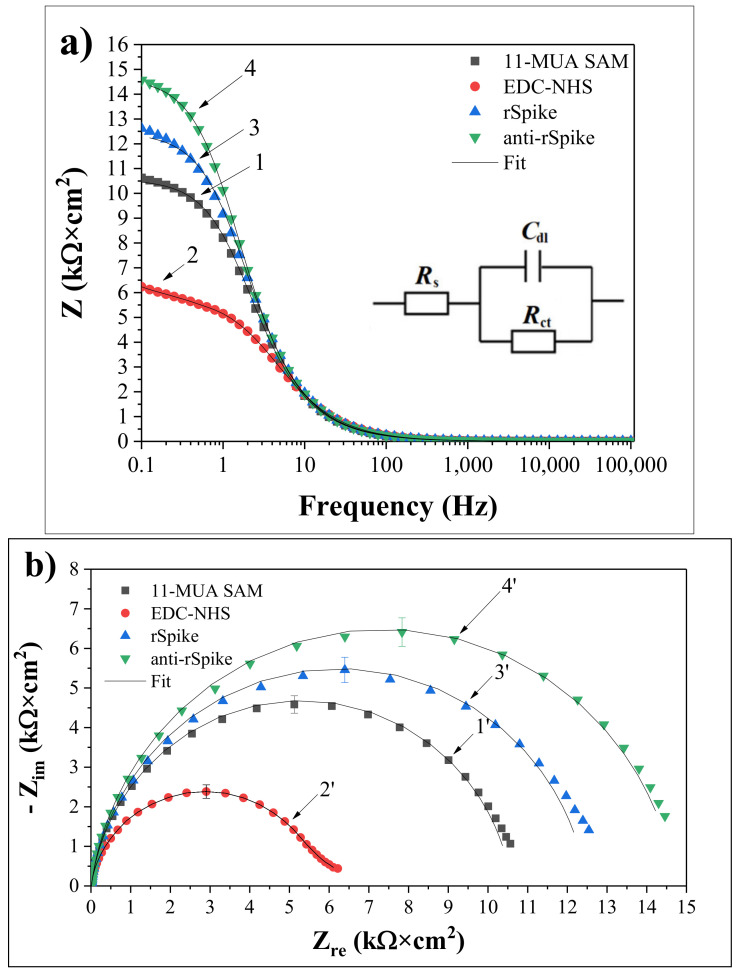
(**a**) Bode plots of differently modified Au electrode: (1) Au/SAM, (2) Au/SAM/EDC-NHS, (3) Au/SAM/rSpike, (4) Au/SAM/rSpike/anti-rSpike. The Randles equivalent circuit was applied for the analysis of EIS data, where R_s_ represents the dynamic solution resistance, C_dl_ is the double layer capacitance measured between the Au electrode and the electrolyte solution, and R_ct_ is the charge transfer resistance of the immobilised recognition layer. (**b**) Nyquist plots of differently modified electrodes: Au electrodes: (1′) Au/SAM, (2′) Au/SAM/EDC-NHS, (3′) Au/SAM/rSpike, (4′) Au/SAM/rSpike/anti-rSpike. EIS measurements were performed in the PBS, pH 7.4, in presence of 2 mM of [Fe(CN)_6_]^3−/4−^ and 0.1 M KCl at 0.2 V vs Ag/AgCl_(3M KCl)_.

EIS was utilized to monitor impedimetric qualities based on the applied equivalent circuit, allowing chemical transformations and processes occurring on the conducting electrode surface to be perceived [42]. Figure 3a shows the impedance responses of the [Fe(CN)_6_]^3−/4−^ based redox probe in PBS on the Au electrode after the formation of the Au/SAM structure based on 11-MUA (Figure 3a-1), activation of SAM with EDC and NHS (Figure 3a-2), covalent immobilization of rSpike (Figure 3a-3), and affinity interaction with anti-rSpike (Figure 3a-4) in the frequency range from 0.1 Hz to 100 kHz.

No significant difference between spectra 1, 2, 3, and 4 is observed (Figure 3a) at frequencies greater than 100 Hz, suggesting that the formation of SAM based on 11-MUA, the immobilisation of rSpike, and the formation of an immunocomplex between rSpike and anti-rSpike (rSpike/anti-rSpike) on the electrode surface did not have any significant impact on the R_s_ value. On the contrary, C_dl_ and R_ct_ are bound to the dielectric and insulating properties of the electrode/electrolyte interface; therefore, they are significantly affected by the changes of the Au-electrode surface. When the frequency of the EIS perturbation decreases, an imaginary component Z_im_ = 1/jC_dl_ becomes important and significantly contributes to the C_dl_ value of the equivalent circuit [43]. The double-layer capacitance (C_dl_) has greater impedance at lower frequencies; as a result of this effect, the current mainly passes through R_ct_ and R_s_. The impedance value of 10.6 kΩ·cm^2^ at a given frequency (0.1 Hz) increased with the formation of the thiol monolayer (Figure 3a-1), the immobilisation of the rSpike (12.6 kΩ·cm^2^) (Figure 3a-3), and the formation of the rSpike/anti-rSpike immunocomplex (14.5 kΩ·cm^2^) (Figure 3a-4) on the surface of the Au electrode, compared to NHS and EDS, the activated Au/SAM/EDC-NHS electrode (6.2 kΩ·cm^2^) (Figure 3a-2). It was observed that the R_ct_ of the Au electrode after the formation of the SAM layer increased and had very low electron transfer efficiency. However, the R_ct_ of the NHS and EDS activated Au/SAM/EDC-NHS electrode considerably decreased compared to that determined before the activation process. The EDC-NHS response involving the development of an intermediate electrochemically active ester was the result of the terminal–COOH interaction with EDS and NHS. The rise in R_ct_ following rSpike and anti-rSpike binding is due to the fact that most proteins have poor electrical conductivity at low frequencies, preventing charge transfer at the electrode-solution interface. These EIS-based data fit well with data presented in our previous investigations, which were based on the evaluation of interactions between SARS-CoV-2 proteins and specific antibodies against these proteins by Total Internal Reflection Ellipsometry, which clearly illustrate that at the interfacial electrode–solution boundary, a significant increase in protein layer thickness and changes of dielectric properties have been observed [6,44].

Despite the fact that it provides the same information as Bode graphs, Nyquist coordinates are ideally suited to depict the electrochemical impedance, especially in the ‘semi-circular area’ of EIS spectra (Figure 3b). As seen in the Figure 3b, the diameter of the semi-circle rises following the formation of SAM based on 11-MUA 4.58 ± 0.22 kΩcm^2^ (Figure 3b-1’), activation of 11-MUA carboxyl groups by EDC and NHS 2.38 ± 0.17 kΩcm^2^ (Figure 3b-2’). Progressive immobilisation of rSpike protein 5.45 ± 0.32 kΩcm^2^ (Figure 3b-3’) and affinity with anti-rSpike 6.41 ± 0.36 kΩcm^2^ (Figure 3b-4’) cause the interphase between the Au electrode and solution to become more insulating, obstructing the passage of charged [Fe(CN)_6_]^3−/4−^ ions and electron exchange between them. As a result, the electron transfer resistance R_ct_ increased as the Au surface was changed step by step, as shown in Figure 3a. The R_ct_ component of different modified electrodes tends to exhibit visible fluctuations, which provide the high sensitivity necessary for the EIS-based approach to detect antigen-antibody complex formation.

## 4. Conclusions

The covalent immobilisation of rSpike and affinity interaction with anti-rSpike were investigated in this work. Cyclic voltammograms revealed that 11-MUA SAM molecules bound and blocked the surface of the Au electrode required for further electron transfer. EIS showed that the charge transfer resistance of the Au/SAM electrode after activation with EDC and NHS decreased when compared with the electrode before activation. The EIS spectra in Nyquist coordinates show distinct changes in each phase of Au electrode modification: the semicircle grows after rSpike immobilisation and the antigen-antibody complex forms after anti-rSpike interaction. This enables the use of impedimetric techniques to detect the antigen-antibody complexes and, as a result, the creation of an immunosensor for the serologic diagnosis of COVID-19 and/or the assessment of vaccination success against the SARS-CoV-2 virus.

## Data Availability

Not applicable.
